# Attosecond spectroscopy reveals spontaneous symmetry breaking in molecular photoionization

**DOI:** 10.1126/sciadv.adw5415

**Published:** 2025-09-19

**Authors:** Mingxuan Li, Leshi Zhao, Huiyong Wang, Jialong Li, Wentao Wang, Jiaao Cai, Xiaochun Hong, Xiaosen Shi, Ming Zhang, Xinning Zhao, Robin Weissenbilder, David Busto, Mathieu Gisselbrecht, Kiyoshi Ueda, Sizuo Luo, Zheng Li, Dajun Ding

**Affiliations:** ^1^ Institute of Atomic and Molecular Physics, Jilin University, Changchun 130012, China.; ^2^ State Key Laboratory for Mesoscopic Physics and Frontiers Science Center for Nano-optoelectronics, School of Physics, Peking University, Beijing 100871, China.; ^3^ Department of Physics, Lund University, Lund 221 00, Sweden.; ^4^ Department of Chemistry, Tohoku University, Sendai 980-8578, Japan.; ^5^ Collaborative Innovation Center of Extreme Optics, Shanxi University, Shanxi 030006, China.; ^6^ Peking University Yangtze Delta Institute of Optoelectronics, Jiangsu 226010, China.

## Abstract

Spontaneous symmetry breaking, driven by nonadiabatic electron-nuclear coupling, can lead to geometric complexity in molecules and solids. While structural distortion from symmetry breaking occurs in femtoseconds, the timescale to lift electronic state degeneracy has remained elusive. We use the vibrationally resolved attosecond chronoscope to capture the electronic symmetry breaking induced by the Renner-Teller effect in bent CO_2_ molecules after photoionization by an extreme ultraviolet photon by measuring attosecond ionization delays. Relative photoionization delays between the four cation states are observed, with vibrational state–dependent delays, we analyze the evolution of the degenerate $A^{2}\Pi_{u}$ state to the nondegenerate *A*′ and *A*″ states due to molecular bending. With the help of theoretical analysis, we show that the relative photoionization delays of up to 72 as between the vibrational levels originate from the symmetry breaking–induced shape resonance. This study offers fundamental insights by resolving the coupled electron and structural dynamics simultaneously.

## Teaser

Vibrationally resolved attosecond photoelectron spectroscopy captures the Renner-Teller symmetry breaking of linear molecules.

## INTRODUCTION

Spontaneous symmetry breaking occurs when a system, originally symmetric under certain transformations, transitions into an asymmetric state. This phenomenon has profound implications across quantum field theory and the standard model of particle physics, such as the Higgs mechanism (*1*). Molecules, with their complex rovibronic modes and potential energy landscapes defined by the atomic composition and spatial arrangement, serve as ideal candidates to study symmetry breaking. The spontaneous symmetry breaking of molecules can be induced by the nonadiabatic coupling between electronic and nuclear degrees of freedom, driven by effects such as Renner-Teller (RT) (*2*), Jahn-Teller (*3*) distortion, and conical intersections (*4*, *5*). These effects signal a breakdown of the Born-Oppenheimer (BO) approximation, which is foundational to our understanding of molecular dynamics and is crucial in a wide array of chemical processes, from molecular reactions to the photostability of DNA (*6*, *7*). In a symmetric molecule, the electronic states can be degenerate because of spatial symmetry breaks when the nuclear motion or electron-nuclear coupling occurs after absorbing photons. The geometric relaxation due to ultrafast symmetry breaking is often tracked through femtosecond spectroscopy that reveals nuclear motion (*8*, *9*); this process can occur on a timescale of less than 10 fs (*10*, *11*). Yet, how the degenerate electronic states drive geometric symmetry breaking affects the attosecond photoemission process remains an open question—one that attosecond spectroscopy may help to answer. The Nobel Prize in Physics (2023) celebrated the breakthroughs in attosecond science (*12*–*14*), underscoring attosecond spectroscopy as a powerful tool to trace electron motion in atoms (*15*–*18*), molecules (*19*–*27*), liquids (*28*, *29*), and solids (*30*–*32*). A key achievement of attosecond spectroscopy is the ability to measure ionization time delays between valence states of atoms and molecules, providing crucial insights into electron correlation (*33*), nuclear motion (*20*, *23*), stereodynamics of molecular ionization (*25*, *26*), molecular environment effect during photoionization (*34*), and the influence of charge distribution of complex molecules (*35*). In molecules, substantial time delays occur during vibrationally dependent photoionization near resonances (*20*, *23*), the influence of stretching motion on photoionization time delay revealing insights into molecular shape resonances and electron correlation effects. Although capturing electron-nuclear coupling dynamics on the attosecond scale is an ongoing challenge of attochemistry (*36*, *37*), it remains key to understanding processes where molecular vibrations are typically frozen, in line with the BO approximation.

**Fig. 1.**
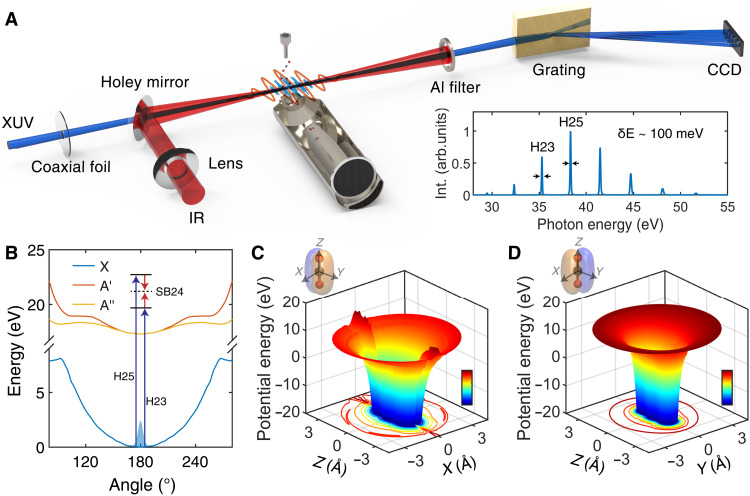
RT effect in photoionization of CO_2_ and its measurement by RABBIT spectroscopy. (**A**) Schematic diagram of the attosecond photoelectron interferometer for RABBIT measurement. The phase-locked XUV–attosecond pulse train pump and IR probe pulse have been used to capture the attosecond time delay in photoionization of CO_2_ molecules; the corresponding HHG spectrum is shown. (**B**) Sketch of the potential-energy curves of ground state and $A^{2}\Pi_{u}$ state in the Franck-Condon region [taken from (*59*, *60*)]; the RT effect causes the degenerate linear $A^{2}\Pi_{u}$ state splitting to *A*′ and *A*″ states as the ${\text{CO}}_{2}^{+}$ cation bends. The two-photon transitions for the formation of SB24 in the RABBIT spectra are illustrated. (**C** and **D**) The potential energy surfaces (PES) of an electron around the $A^{2}\Pi_{u}$ state of a symmetry broken ${\text{CO}}_{2}^{+}$ cation, which bends in the *OYZ* plane; the potential is plotted in two orthogonal planes, i.e., the *OXZ* plane for the *A*′ state and the *OYZ* plane for the *A*″ state; the contour lines depict the external shape of the PES.

RT distortion represents a fundamental mechanism that spontaneously reduces the symmetry of linear polyatomic molecules such as ${\text{CO}}_{2}^{+}$ and lifts the degeneracy of electronic states upon bending (*2*, *38*, *39*), which can be more evident during multimode vibronic coupling (*40*). Synchrotron radiation spectroscopic studies have extensively explored its photoionization process, where ionization cross sections and asymmetry parameters can be provided by experimental measurements and theoretical calculations (*41*–*45*). Recent attosecond spectroscopy addressed the impact of electron correlation (*46*) and interchannel coupling (*47*) between cation states on linear geometry. However, the correlation between spontaneous symmetry breaking and the attosecond photoionization process in the nonadiabatic regime is not established at present. In this study, we use vibrational state–resolved RABBIT (reconstruction of attosecond beating by interference of two-photon transitions) spectroscopy (*13*, *48*) to explore the spontaneous symmetry breaking of the ${\text{CO}}_{2}^{+}$ cation as shown in Fig. 1. Specifically, the recombined extreme ultraviolet (XUV) and infrared (IR) pulses are focused onto CO_2_ molecules from a gas jet, leading to the preparation of bent molecular cations via vertical transitions following XUV photon absorption from the neutral molecules. The photoionization delay of CO_2_ molecules along the laser polarization is captured by RABBIT measurements using a field-free electron time of flight in Fig. 1A. The electronic symmetry breaking arises from the nonadiabatic electronic-nuclear coupling induced from the RT effect, occurring while the molecular structure remains effectively frozen on such an ultrashort timescale. The RT effect manifests in ${\text{CO}}_{2}^{+}$ after bending when the $A^{2}\Pi_{u}$ state is populated following ionization of CO_2_ by an XUV photon as shown in Fig. 1B. Thus, the two-photon photoionization delay, which is sensitive to the cation’s potentials (in Fig. 1, C and D), can be extracted by RABBIT measurements. The potential energy surface of the *A*′ state observed from the *OXZ* plane (Fig. 1C) and that of the *A*″ state observed from the *OYZ* plane (Fig. 1D) substantially differ with each other after the RT splitting of the $A^{2}\Pi_{u}$ state, leading to a variation in the ionization time delay and providing information on electron-nuclear coupling dynamics.

## RESULTS

### RABBIT spectroscopy of CO_2_ molecules

**Fig. 2.**
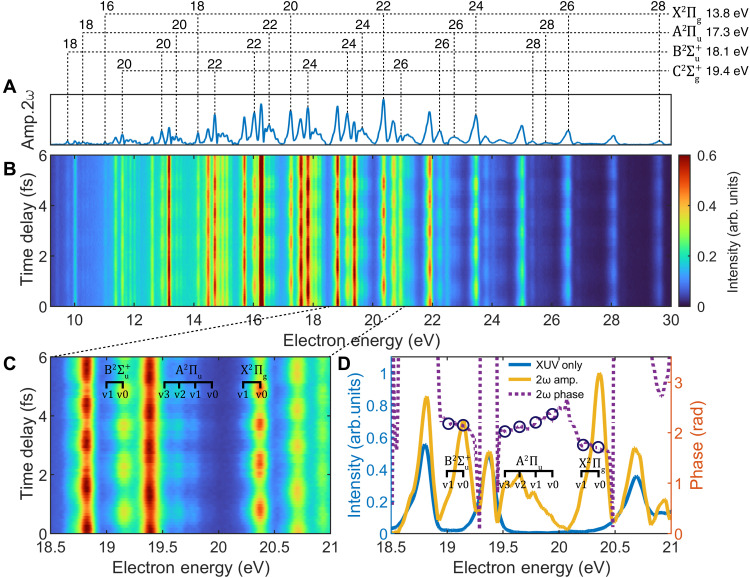
Channel-resolved CO_2_ RABBIT spectra. (**A**) Identification the SB channels of four cation states ( $X^{2}\Pi_{g}$ , $A^{2}\Pi_{u}$ , $B^{2}\Sigma_{u}^{+}$ , and $C^{2}\Sigma_{g}^{+}$ ) with the 2ω amplitude spectrum. (**B**) Full XUV-IR spectrum as a function of the delay. (**C**) The vibrational state–resolved RABBIT spectrum, labeled with the positions of SB22 for $X^{2}\Pi_{g}$ and SB24 for $A^{2}\Pi_{u}$ , $B^{2}\Sigma_{u}^{+}$ states. (**D**) The XUV-only (blue), 2ω amplitude (orange), and 2ω phase (purple dashed) spectra.

We have measured attosecond ionization time delays that cover the first four electronic states ( $X^{2}\Pi_{g}$ , $A^{2}\Pi_{u}$ , $B^{2}\Sigma_{u}^{+}$ , and $C^{2}\Sigma_{g}^{+}$ ) of ${\text{CO}}_{2}^{+}$ , along with the resolution of the vibrational levels. The experiment uses XUV photons with energies of 26 to 45 eV, 17th to 29th harmonics from high harmonic generation (HHG) driven by 800-nm femtosecond laser interaction with argon gas that forms attosecond pulse trains. Our measurements present a notable ionization delay due to shape resonance in the $C^{2}\Sigma_{g}^{+}$ state. More intriguingly, we capture relative attosecond ionization time delays between vibrational levels (*v* = 0 to 3) for the $A^{2}\Pi_{u}$ state, which originate from spontaneous symmetry breaking–induced shape resonance. This provides a unique window into the coupling between vibrational motion and electronic symmetry breaking processes in molecules, highlighting the sensitivity of attosecond spectroscopy in probing the interplay of electron-nuclear coupling. We investigate the attosecond molecular ionization with the RABBIT spectroscopy of CO_2_ with high temporal and energy resolution (≤20 as, ≤100 meV). This approach allows us to resolve both the electronic and vibrational levels of the molecule (fig. S1). The channel-resolved RABBIT spectra, depicted in Fig. 2, are obtained from delay-dependent photoelectron spectra measurements that show oscillations in signal intensity. These oscillations induced from two-path two-photon interference, where the absorption of one harmonic and an IR photon, along with the absorption of the next harmonic and emission of an IR photon, lead to the same final energy and the interference generates oscillations in the sideband (SB) intensities. The oscillation follows the form

$$S\left(\tau \right)=\alpha +\beta \text{cos}\left[2\omega \left(\tau -\tau_{\text{XUV}}-\tau_{\text{mol}}\right)\right]$$ (1)

where α and β are delay-independent constants, ω is the IR laser carrier frequency, $\tau_{\text{XUV}}$ is the attosecond chirp of the XUV light, and $\tau_{\text{mol}}$ represents the ionization time delays of molecules during two-photon ionization.

In Fig. 2A, the 2ω amplitude spectrum after fast Fourier transform (FFT) along the time delay axis of RABBIT spectroscopy (shown in Fig. 2B) resolves signals corresponding to the $X^{2}\Pi_{g}$ , $A^{2}\Pi_{u}$ , $B^{2}\Sigma_{u}^{+}$ , and $C^{2}\Sigma_{g}^{+}$ cation states and their vibrational levels from SB18 to SB28. Figure 2C zooms into the 18.5- to 21-eV energy range, marking SB signals for the $X^{2}\Pi_{g}$ , $A^{2}\Pi_{u}$ , and $B^{2}\Sigma_{u}^{+}$ states, including four vibrational levels (*v* = 0 to *v* = 3) for the $A^{2}\Pi_{u}$ state at SB24. An energy-resolved RABBIT analysis is shown in Fig. 2D, capturing the amplitude and phase for each cation state and vibrational level, with vibration-dependent phases marked for $A^{2}\Pi_{u}$ as shown in purple dashed curve. For identification, the signal from XUV only is also plotted in the blue curve. There is no overlap between the SB signals from the $X^{2}\Pi_{g}$ , $A^{2}\Pi_{u}$ , and $B^{2}\Sigma_{u}^{+}$ states and the XUV-only signal, as shown in the difference between the orange and blue curves. Additional comparisons of the photoelectron spectra obtained using XUV pulse and XUV combined with IR pulses are shown in fig. S1. These comparisons enable reliable extraction of the photoionization delays, with results from three different methods summarized and discussed in fig. S2. Notably, the extracted vibration-dependent phases—highlighted as blue circles—exhibit pronounced variations for the $A^{2}\Pi_{u}$ vibrational levels at SB24, a feature not observed in other SBs (additional analysis of SB20 is provided in fig. S3). Moreover, extended RABBIT scans acquired under varied experimental conditions are shown in fig. S4.

### Attosecond photoionization time delays between cation states

**Fig. 3.**
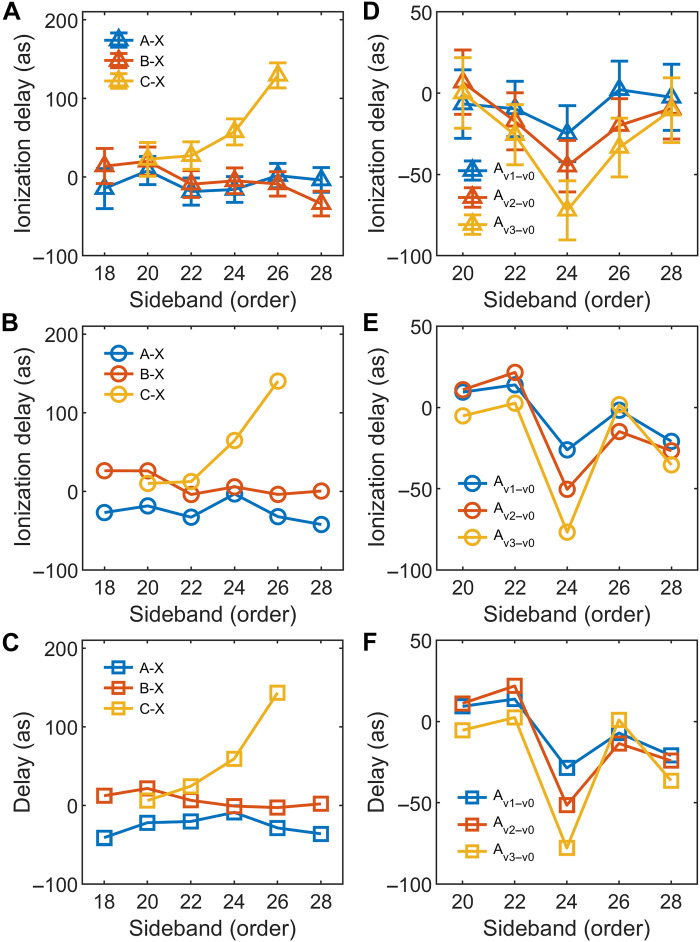
Photoionization delays of cations. (**A** to **C**) Extracted the first four cation states’ photoionization delay from the experiment (triangle), as well as the two-photon (circle) and one-photon (square) ionization delay from the theory, respectively, using the ionization delay of $X^{2}\Pi_{g}$ as a reference in each case. (**D** to **F**) The vibrational-resolved relative delay within $A^{2}\Pi_{u}$ from the same experiment and theory, using the ionization delay of A_v0_ as a reference.

Attosecond photoionization time delays between four final cation states ( $\tau_{X,A,B,C}$ ) were extracted by comparing delay differences between the $X^{2}\Pi_{g}$ , $A^{2}\Pi_{u}$ , $B^{2}\Sigma_{u}^{+}$ , and $C^{2}\Sigma_{g}^{+}$ states from the same harmonics. The $X^{2}\Pi_{g}$ state was chosen as the reference because of the absence of shape resonance in this photon energy regime (*44*). Relative time delays $\tau_{A-X}$ , $\tau_{B-X}$ , and $\tau_{C-X}$ were extracted using cosine fitting and are plotted in Fig. 3A, and the methodology used can be seen in the Supplementary Materials. A positive delay of 129 ± 15 as at SB26 (~40.3 eV) was observed for the $C^{2}\Sigma_{g}^{+}$ state, arising from a well-known σ*_u_* shape resonance near 42 eV caused by the partial wave *l* = 5 (*44*). This delay is consistent with trapping an electron in a Coulomb potential induced by shape resonance, as described in (*20*). We calculated the relative two-photon ionization delays between the four cationic states, incorporating both interchannel coupling and continuum-continuum (CC) transitions. The results are presented in Fig. 3B. The one-photon Wigner delays ( $\tau_{\text{Wigner}}$ ) for the four cation states were computed using the ePolyScat code (*49*, *50*), which uses a close-coupling expansion of the scattering wave function to account for multichannel configuration interactions between cationic states (see the Supplementary Materials for details). The corresponding Wigner delays are shown in Fig. 3C. The photon energy–dependent CC delay, $\tau_{\text{CC}}$ , was calculated using a previously established formula developed for molecular systems (*51*). The relative Wigner delays between the $A^{2}\Pi_{u}$ , $B^{2}\Sigma_{u}^{+}$ and $X^{2}\Pi_{g}$ states did not show notable variation and agreed with the measurements, as expected from the absence of resonance in this energy regime. However, a large ionization delay of 140 as for $C^{2}\Sigma_{g}^{+}$ at SB26 was observed as shown in Fig. 3B, in good agreement with experimental results. In the meanwhile, the relative Wigner delay between four cation states shows the same trends with the measurements, and $\tau_{\text{CC}}$ only gives a minor contribution to the photoionization delay in this energy region. The pronounced relative Wigner delay of approximately 143 as for the SB26 of $C^{2}\Sigma_{g}^{+}$ state arises from the electron’s interaction with the shape resonance potential.

### Vibrationally resolved photoionization time delays

The vibrationally resolved photoionization time delays for $A^{2}\Pi_{u}$ were extracted by integrating the energy intervals for *v* = 0, 1, 2, and 3 (Fig. 3D). Details regarding the energy intervals and the data extraction procedure are provided in the Supplementary Materials. The relative ionization time delays vary with vibrational levels, from −25 as for *v* = 1 to −72 as for *v* = 3 at SB24 (~37.2 eV), and becomes near zero by SB28. Here, the ionization delay of *v* = 0 is set as a reference. The vibrational state sensitivity of the ionization time delays illustrates the potential of attosecond spectroscopy for studying electron-nuclear coupling. Moreover, the relative ionization delay between *v* = 1 and *v* = 0 for $X^{2}\Pi_{g}$ state exhibits no notable energy dependence and remains close to zero across SB20 to SB28. For the nonadiabatic coupling of electron dynamics with different vibrational levels of $A^{2}\Pi_{u}$ state, we find the vibrationally resolved transition probability between the initial state and the final state as

$$\begin{array}{c} P_{\text{gs}\to A,v}\left(Q_{g},\rho ,E\right)={|\varphi_{\text{gs}}\left(Q_{g},\rho \right)\cdot d_{A}(Q_{g},\rho ,E)\cdot \varphi_{A,v}(Q_{g},\rho )|}^{2} \end{array}$$ (2)

where *Q_g_* denotes the symmetric stretching coordinate, ρ is the bending amplitude, the subscript gs represents the ground state, *E* is the kinetic energy of the outgoing electron, φ represents the vibrational wave functions that are calculated using the multiconfigurational time-dependent Hartree method (*52*–*55*), and *d_A_* is the transition matrix element calculated using the ePolyScat code. The vibrational levels with quanta in the symmetric stretching and bending modes were found to be mainly populated in the $A^{2}\Pi_{u}$ state (*45*), so we ignore asymmetric stretching in the calculation. We obtain the representative molecular configurations in $C^{2}\Sigma_{g}^{+}$ , $B^{2}\Sigma_{u}^{+}$ , $X^{2}\Pi_{g}$ and the vibrational levels ( v=0,1,2,3 ) of the $A^{2}\Pi_{u}$ state from the probability $P_{\text{gs}\to A,v}$.

We have performed the two-photon ionization calculation with the interchannel coupling included under representative molecular configurations. The calculated relative photoionization delays between the four vibrational levels are shown in Fig. 3E. The calculated results agree well with the measured data shown in Fig. 3D, with consistent energy and vibrational level dependence from SB20 to SB28. The relative ionization time delays are calculated to be −26, −50, and −77 as for $\tau_{v1-v0}$ , $\tau_{v2-v0}$ , and $\tau_{v3-v0}$ at SB24, which are quantitatively consistent with the measured data within an accuracy of 5 as. Furthermore, the corresponding relative Wigner delays are shown in Fig. 3F. The relative $\tau_{\text{CC}}$ among the four vibrational levels contributes negligibly to the measured ionization delays and is therefore beyond the scope of the present discussion. We also performed calculations excluding interchannel coupling, which yielded relative Wigner delays that retain the same dependence on photon energy and vibrational level. These results indicate that an alternative interaction mechanism must be considered to understand the observed ionization delays during photoemission process.

### The effect of RT symmetry breaking

**Fig. 4.**
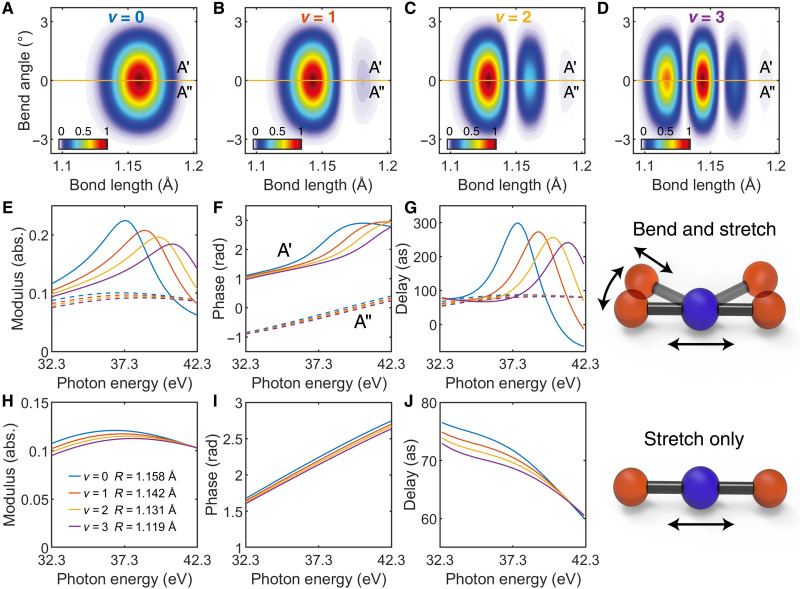
Interpretation of the geometry dependence time delay of vibrational levels in CO_2_. (**A** to **D**) Transition probability from initial ground state to the final vibrational levels *v* = 0, 1, 2, and 3 of $A^{2}\Pi_{u}$ , respectively, on *A*′ (top) and *A*″ (bottom) state. (**E** to **G**) The transition matrix elements analysis, amplitude, phase, and Wigner delay, for *A*′ (solid lines) and *A*″ (dashed lines) states under the molecular configuration with 1° bending angle and 1.158 Å (*v* = 0, blue), 1.142 Å (*v* = 1, red), 1.131 Å (*v* = 2, orange), 1.119 Å (*v* = 3, purple) R_CO_, respectively. (**H** to **J**) Same transition matrix analysis but with linear molecular configuration and $A^{2}\Pi_{u}$ state.

Our investigation unveils the relation between the attosecond ionization time delay and the RT effect, which will bend the linear geometry and split the degenerate $A^{2}\Pi_{u}$ state into two nondegenerate *A*′ and *A*″ states of the ${\text{CO}}_{2}^{+}$ cation (see the Supplementary Materials for details). We conducted an analysis of ionization time delays for the four vibration levels in the symmetry broken *A*′ and *A*″ states. The transition probability from the molecular ground state to four vibrational levels of *A*′ and *A*″ states by XUV photoionization is calculated and given in Fig. 4 (A to D). In the photoionization calculation, the representative geometries preserve bent structures as the RT effect is included, with a bending angle of 1°. The bond length R_CO_ for the C═O bonds are taken to be 1.158, 1.142, 1.131, and 1.119 Å for *v* = 0 to *v* = 3. We have calculated the cross sections and the scattering phases for four vibration levels in the *A*′ and *A*″ states and compare with the results from the linear geometries of $A^{2}\Pi_{u}$ state without the RT effect, as depicted in Fig. 4 (E to J). The results show that in the 32.3- to 42.3-eV energy regime, the cross sections, scattering phase, and Wigner delay for the bent *A*″ state are similar to that of the linear $A^{2}\Pi_{u}$ state, with no apparent signature of resonance. However, the cross sections and scattering phases for the four representative geometries in the *A*′ state show evidence of resonances, which lead to strong scattering phase variation and are very sensitive to the R_CO_ bond lengths. The potential felt by the emitted photoelectron from *A*′ and *A*″ states is different, as shown in Fig. 1 (C and D), and the electron can be trapped in the dip barrier of the *A*′ potential. This behavior resembles the shape-resonance potential of molecules, and the observed dependence on bond length aligns with previous findings related to molecular shape resonance (*20*, *23*).

The calculated Wigner time delays, $\tau_{\text{Wigner}}$ , for four R_CO_ bond lengths in the bent *A*′, *A*″ states, and the linear $A^{2}\Pi_{u}$ state are presented in Fig. 4 (G and J). The relative Wigner time delays between the four vibration levels in the $A^{2}\Pi_{u}$ state are less than 5 as in our energy regime, which is out of current measurement precision. The differences in attosecond Wigner time delays are found for the four vibrational levels in the *A*′ state (see partial wave analysis in figs. S5 to S7), which stands as evidence that the RT distortion induces the time delay difference for the vibrational levels. While there is no similar feature in bent *A*″ state and linear $A^{2}\Pi_{u}$ state, more details about partial wave analysis are in the Supplementary Materials. The Wigner time delay varies from tens of as to approximately 300 as in this energy regime for *A*′ state, providing evidence of spontaneous symmetry breaking, as shown in Fig. 4G.

The partial wave analysis reveals shape resonance in cross sections for odd partial waves, in which the *l* = 1 component dominates the contribution. In addition, the energy of the cross-section maxima is highly dependent on the bond length R_CO_ for the *A*′ state, and no signature of resonance is observed for the *A*″ state. For comparison, we have performed calculations for the symmetric stretch-only geometries in the linear configuration, and the results are presented in Fig. 4J. It shows that only minor relative Wigner time delays exist between the four vibration levels if no symmetry breaking–induced resonance participates. Therefore, our finding here highlights the critical role of bending distortion in the Wigner time delays, which forms a direct link between the attosecond delay and the profound spontaneous symmetry-breaking process. We show that the geometric bending of the ${\text{CO}}_{2}^{+}$ cation is the underlying cause for the appearance of vibrational state dependence of time delays in the measured energy regime around SB24. To observe this phenomenon in the photoionization time delay measurements, as did in Fig. 3C, the degeneracy of the linear $A^{2}\Pi_{u}$ state must be lifted before the electron propagates through half of the molecular Coulomb potential. Thus, the time scale of the lift of degeneracy should be faster than the Wigner time delay. In this way, we demonstrate that attosecond spectroscopy captures the signature of spontaneous electronic symmetry breaking induced by RT distortion in the Wigner time delay. Furthermore, the Wigner delay is highly sensitive to molecular bending in cases of RT-type spontaneous symmetry breaking, which indicates that attosecond photoelectron spectroscopy reveals the electron-nuclear coupling dynamics with very high accuracy. Specifically, as the bending angle increases from 0.5° to 2°, the Wigner delay rises from less than 200 as to nearly 450 as, as can be seen in fig. S8.

## DISCUSSION

To conclude, attosecond photoelectron interference spectroscopy provides a sensitive probe of the RT effect–induced spontaneous symmetry breaking in the photoionized ${\text{CO}}_{2}^{+}$ cation. The relative photoionization time delays between four vibrational levels in the $A^{2}\Pi_{u}$ state of cation are measured and analyzed with theoretical calculations. We find that the resonance of the *A*′ state, induced by electronic symmetry breaking under a bent molecular geometry, is responsible for the observed ionization delays among the four vibrational levels. These results demonstrate the remarkable sensitivity of attosecond spectroscopy in capturing subtle structural distortions in the degenerate $A^{2}\Pi_{u}$ state arising from nonadiabatic electronic-nuclear coupling, such as a bond stretching of 0.04 Å under bending angle of 1° will advance the ionization delay by 72 as. Moreover, our observations give a strong indication that the degeneracy of the electronic state occurs on a timescale faster than the Wigner time delay. Our findings reveal profound insights into photoionization time delays stemming from nuclear-electronic coupling, opening pathways for studying symmetry-breaking dynamics in nonadiabatic effects in photon-matter interactions.

## MATERIALS AND METHODS

### Experimental methods

The high-order harmonic generation is driven by an IR laser (1 kHz, 800 nm, 35 fs, and 7 mJ) provided by a Ti:Sapphire laser system. The IR laser was initially split into two beams (R/T = 2:8) with an actively stabilized Mach-Zehnder interferometer for the attosecond controlled pump-probe scan. The strongest part in the pump arm (approximately 3 × 10^14^ W/cm^2^) is focused on a custom-built gas cell by a spherical mirror (Ag-coated, *f* = 500 mm), where it interacts with argon gas to generate narrow-band HHG emission. After passing through a coaxial filter and a holey mirror, the central part of IR beam is filtered out, while the XUV beam is focused using a toroidal mirror (Au-coated, *f* = 700 mm). The weaker portion of the IR beam is focused by a lens (*f* = 1000 mm) in the probe arm with intensity ~2 × 10^11^ W/cm^2^ for the RABBIT measurement. The pump-probe delay in this attosecond interferometer is feedback controlled by a piezo stage based on the interference fringe of two reference copropagate 532-nm continuous wave laser beams, ensuring long-term stability with a root mean square below 20 as (*56*). Subsequently, the RABBIT measurements were performed by scanning the XUV-IR delays and collecting the electrons. The CO_2_ gas target is injected through a stainless steel capillary (100-μm diameter) to interact with the focused XUV and IR beams. During the measurements, a field-free time-of-flight spectrometer ( E/ΔE≥100 @ 10 eV) collects the ionized electrons. The photoionization delays are extracted using three methods: FFT, cosine fitting, and complex fitting (*28*, *57*). All three approaches yield consistent results, with good agreement among the extracted values. Further details are given in the Supplementary Materials.

### Theoretical methods

Around the equilibrium configurations of the CO_2_ molecule, we compute the molecular orbitals using the MOLPRO package with valence triple zeta basis set for the different molecular configurations (different C═O bond lengths and molecular bond angles) (*58*). Using molecular orbitals as input, we use ePolyScat to calculate the dipole matrix elements for different molecular configurations (*49*, *50*). ePolyScat calculates the scattered photoelectron wave function based on the Schwinger variational method. We use Eq. 2 to calculate the transition probability under different molecular configurations and to obtain the representative molecular configurations for the $C^{2}\Sigma_{g}^{+}$ , $B^{2}\Sigma_{u}^{+}$ , $X^{2}\Pi_{g}$ states and the vibrational levels ( v=0,1,2,3 ) of the $A^{2}\Pi_{u}$ state. We calculate the two-photon ionization matrix element by referring to (*51*). In addition, we considered the influence of interchannel coupling with a close-coupling expansion (*23*, *46*). We obtain the two-photon ionization delay of a photoelectron SB corresponding to energy 2*q*ω in the finite-difference approximation. See more details in the Supplementary Materials. To compare with the experimental results, where only the photoelectrons flying along the polarization direction of the ionized light can be collected by the field-free time-of-flight spectrometer, we consider the preferences ionization and numerically calculate the two-photon ionization delay in case of the molecular orientation is near the polarization direction of the ionized light. See more details in the Supplementary Materials.

## Supplementary Materials

### This PDF file includes:

Supplementary Text Figs. S1 to S9 Tables S1 and S2 References
