# Supplementary material for: Attosecond spectroscopy reveals spontaneous symmetry breaking in molecular photoionization
Source: Sci Adv. 2025 Sep 19;11(38):eadw5415. doi: 10.1126/sciadv.adw5415 (PMC12448068; doi:10.1126/sciadv.adw5415)
Supplement: Supplementary file 1 — Supplementary Text Figs. S1 to S9 Tables S1 and S2 References [file sciadv.adw5415_sm.pdf]

Science Advances
AAAS

Supplementary Materials for

Attosecond spectroscopy reveals spontaneous symmetry breaking in molecular photoionization

Mingxuan Li *et al.*

Corresponding author: Sizuo Luo, luosz@jlu.edu.cn; Zheng Li, zheng.li@pku.edu.cn; Dajun Ding, dajund@jlu.edu.cn

Sci. Adv. **11**, eadw5415 (2025)
DOI: 10.1126/sciadv.adw5415

This PDF file includes:

Supplementary Text
Figs. S1 to S9
Tables S1 and S2
References

Supplementary Text

Related spectra in channel resolved RABBIT

Based on the narrowband harmonic source and the high-resolution spectrometer, we can measure the first four electronic states of carbon dioxide unambiguously in XUV-only (blue line) and XUV+IR (red line) spectra, and the results are shown in Fig. S1. The inset figure zoomed in 12.8 to 16 eV, which shows that the $A^2\Pi_u$ state has a clear vibrational state resolution from v = 0 to v = 4. However, the SB of vibrational state v = 4 will overlap with the MB of $C^2\Sigma_g^+$ state in the RABBIT measurements, as shown in Fig. S1B-D with multi-Gaussian fitting, and therefore we mainly discuss the vibrational levels from v = 0 to v = 3.

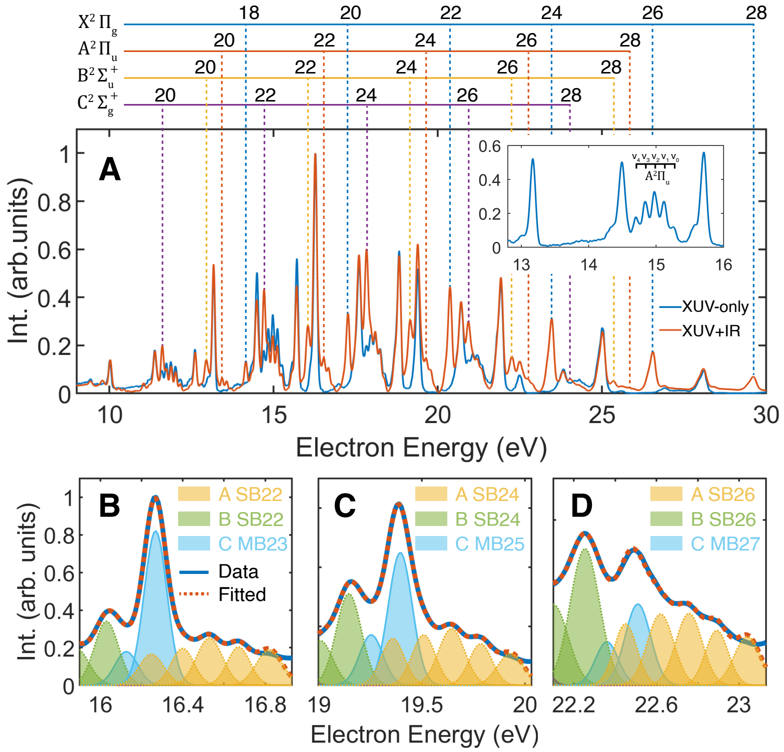

Figure S1: **Photoelectron spectra.** (**A**) The XUV-only (blue line) and XUV+IR (red line) photoelectron spectra of CO_2. And the inset is a zoomed-in view of the energy interval from 12.8 to 16 eV, where the vibrational levels from v = 0 to v = 3 can be well resolved in two-photon ionization case. Experimental measured data (blue solid line) and multi-gaussian fitted data (red dashed line and colored peaks) in the (**B**) SB22, (**C**) SB24 and (**D**) SB26 energy region of $A^2\Pi_u$ state.

Furthermore, we applied *Complex Fitting analysis* (*28, 57*) to distinguish between different channels corresponding to: (1) ionization processes (XUV-only vs. XUV+IR) and (2) final electronic/vibrational levels (v = 0-1 for X/B/C states; v = 0-4 for A state).

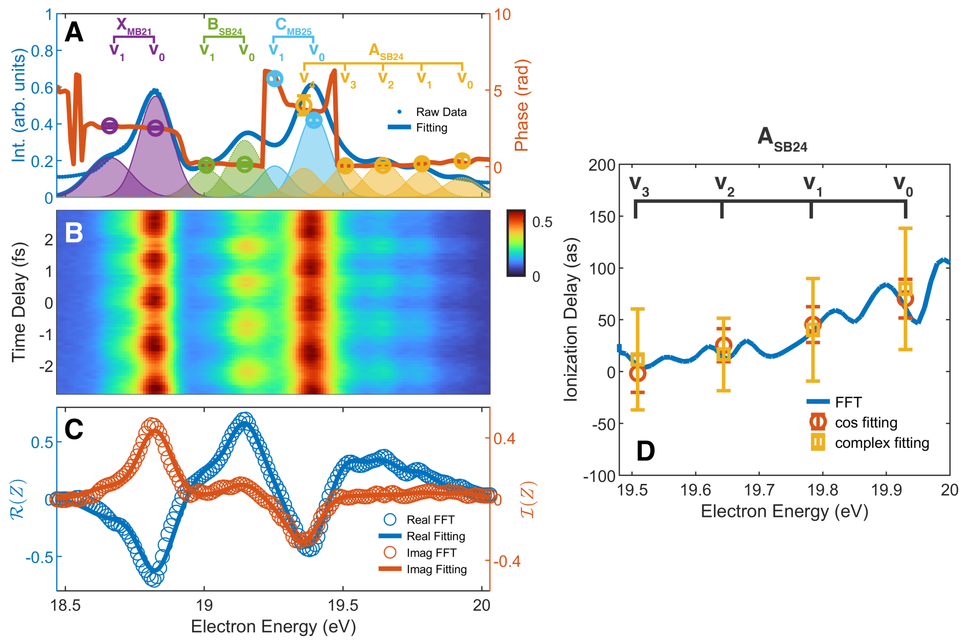

Figure S2: **Complex fitting analysis.** (A) Photoelectron spectra (blue dots) and the corresponding multi-Gaussian fitting (blue line). The FFT phase (red line) and extracted phases (colored circles) for MB21 of X state , SB24 of A stateand B state, MB25 of C state. The colored discrete peaks and extracted phases of same electronics states are shown. (B) Experimental RABBIT spectra. (C) Raw data (circle) and fitted data (lines) of the real (blue) and image (red) part of the complex matrix. (D) Comparison of ionization delay from three phase extraction methods: FFT (blue line), cos fitting (red circle) and complex fitting (orange square).

In this analysis, we first perform multi-Gaussian fitting in Fig. S1B-D based on both XUV-only and XUV+IR spectra to extract the characteristic peak information $p_j(E)$ - including amplitude, center position, and width parameters - for each ionization channel. Subsequently, we can isolate the phase information of individual peaks through fitting of the complex matrix $I(E)$, derived from Fourier transformation of the RABBIT traces along the time-delay axis.

$$I(E) = \sum_j p_j(E) e^{z_j} = \sum_j \underbrace{e^{a_j} p_j(E)}_{A_j(E)} e^{ib_j}. \tag{S1}$$

Where $A_j(E)$ and b_j are Fourier amplitude and phase. The present methodology effectively eliminate the phase distortions induced by spectral overlap, enabling precise isolation of channel-specific phase information. However, for channels exhibiting severe spectral overlap (particularly the v = 4 of A state), the retrieved information remains ambiguous. Accordingly, our subsequent analysis is restricted to the well-characterized $v = 0$ to $v = 3$ vibrational levels

The detailed analysis results are presented in Fig. S2, where (A) displays the discrete peaks (colored peaks) and decoupled phases (colored circles) of different vibrational channels, (B) shows raw RABBIT spectra same in the main text, and (C) illustrates the real (blue) and imaginary (red) parts of the complex FFT matrix, with experimental data represented by circles and fitted results by solid lines. The extracted relative time delay are -68 as (v_3-v_0), -63 as (v_2-v_0) and -39 as (v_1-v_0), showing close alignment and a consistent trend with the values reported in the main text. The comparison of the results shown in (D) obtained from three different methods, FFT, cos fitting and complex fitting, demonstrate that spectral overlap has minimal effect on the determination of photoionization delay.

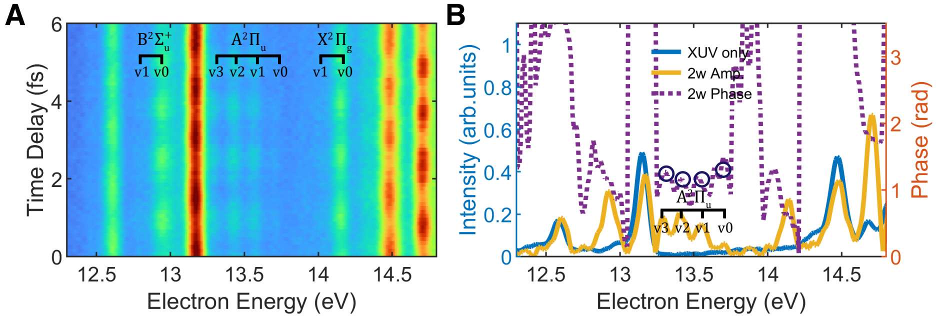

Figure S3: **Channel-resolved RABBIT spectra of** $A^2\Pi_u$ **state. (A)** The vibrational state-resolved RABBIT spectrum, labeled with the positions of SB18 for $X^2\Pi_g$ and SB20 for $A^2\Pi_u$, $B^2\Sigma_u^+$ states. **(B)** The XUV-only (blue), 2ω amplitude (orige) and 2ω phase (purple dashed) spectra.

In addition, in comparison with SB24 of $A^2\Pi_u$ state in the main text, we also extracted the spectral information of SB20 as shown in Fig. S3. In this energy interval, the spectral resolution is sufficient for vibrationally resolved measurements of the $X^2\Pi_g$, $A^2\Pi_u$, and $B^2\Sigma_u^+$ states. Unlike the

large variation with the vibrational quantum number in the SB24 interval, there is no notable phase difference at this energy for different vibrational levels of $A^2\Pi_u$ electronic state.

RABBIT data under different acquisition conditions

To ensure the quality of data presented in the main text, we have repeated the measurements under different acquisition conditions, i.e., step size, acquisition time, and scan range. Under those parameters, we scanned three groups under 120s/150as/25fs, 300s/150as/6fs, and 600s/100as/6fs, respectively. The RABBIT spectra are shown in Figs. S4A-C, and the extracted relative ionization delays for different vibrational levels (Figs. S4D-F) all with the identical trend, and it is notable that the high counts in the 600s/100as/6fs group bring about a notable error reduction, which is chosen to be represented in the main text.

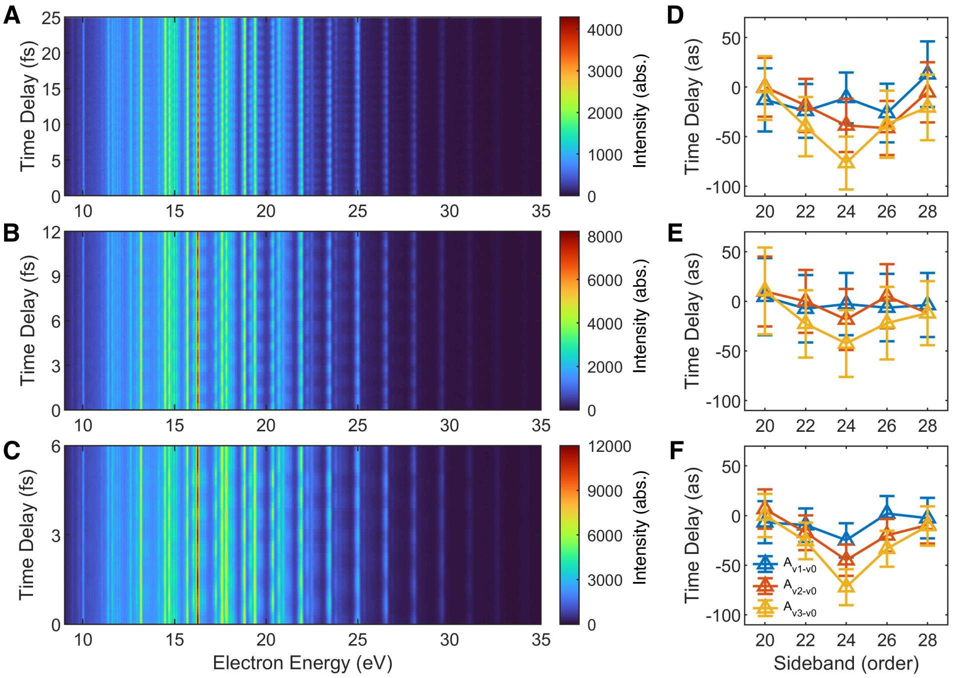

Figure S4: **Experimental spectra and measured time delay under different scan parameters. (A-C)** RABBIT spectra; **(D-F)** relative time delay for different vibrational levels of $A^2\Pi_u$ state. The different acquisition conditions (the integrate time, scan size, and scan range) are 120s/150as/25fs, 300s/150as/12fs, and 600s/100as/6fs, respectively.

Phase extraction methodology

In Fig. 2D of the main text, the phase curve (purple dashed) was derived through Rainbow RABBIT analysis, a technique involving the application of Fast Fourier Transform (FFT) to each individual energy pixel across the photoelectron spectrum. The resulting phase curve facilitates for direct visualization of phase variation as a function of electron energy. The quantitative analysis of the experimental data in Figs. 3A and 3D employed a dual-scale spectral integration strategy coupled with cosine fitting, the energy range are shown in the Tables. S1 and S2. The electronic-state integration spanned ±0.15 eV for $X^2\Pi_g/B^2\Sigma_u^+$ states (v = 0 to 1), ±0.25 eV for $A^2\Pi_u$ state (v = 0 to 3), and ±0.10 eV for $C^2\Sigma_g^+$ state (v = 0), optimized to maximize Franck-Condon profile fidelity while reducing neighboring-state distortion. Concurrent vibrational analysis utilized ±0.04 eV windows to resolve individual transitions without compromising signal integrity.

Table S1: Integrate energy range for the first four electronic states of CO_2.

Electronic	$X^2\Pi_g$	$A^2\Pi_u$	$B^2\Sigma_u^+$	$C^2\Sigma_g^+$
Δ E (eV)	0.15	0.25	0.15	0.10

Table S2: Integrate energy range for the vibrational levels of $A^2\Pi_u$ state.

Vibrational	v_0	v_1	v_2	v_3
Δ E (eV)	0.04	0.04	0.04	0.04

Theory of photoionization delay calculation in ePolyScat

We can use the ePolyScat code to determine the photoionization dipole matrix element for a specified photon energy (*49, 50*). The ePolyScat code uses the Schwinger variational technique. The Schrödinger equation for the wave function $\Psi_{\vec{k}}(\vec{r})$ of the outgoing photoelectron with the momentum $\vec{k}$ (in atomic units) is

$$(-\frac{1}{2}\nabla^2 + V(\vec{r}) - \frac{|\vec{k}|^2}{2})\Psi_{\vec{k}}(\vec{r}) = 0\,, \tag{S2}$$

where $V(\vec{r})$ is the static exchange potential (*50*). Using spherical harmonic function expansion with angular quantum numbers l and m to solve the Eq. S2, the dipole matrix elements in length form can be obtained as follows

$$\mathcal{I}^{(\mathrm{L})}_{l,m,\vec{e}_\mathrm{p}} = (|\vec{k}|)^{\frac{1}{2}} \langle \Psi_\mathrm{i} | \vec{r} \cdot \vec{e}_\mathrm{p} | \Psi_{\vec{k},lm} \rangle \,, \tag{S3}$$

and the dipole matrix elements in velocity form is

$$\mathcal{I}^{(\mathrm{V})}_{l,m,\vec{e}_\mathrm{p}} = \frac{(|\vec{k}|)^{\frac{1}{2}}}{E_\mathrm{p}} \langle \Psi_\mathrm{i} | \nabla \cdot \vec{e}_\mathrm{p} | \Psi_{\vec{k},lm} \rangle \,, \tag{S4}$$

where Ψ_i is the initial state, $\Psi_{\vec{k},lm}$ is the wave function of photoelectron, $\vec{e}_\mathrm{p}$ is the direction of the linearly polarized light and E_p is the photon energy (*50*).

In the process of ionization, we focus on the dipole interaction between a molecule and ionizing light, where the coupling between light and matter is

$$\mathcal{D}(E_\mathrm{e}) = \langle \Psi_\mathrm{f}; \Psi_{\vec{k}} | \vec{\mu} \cdot \vec{E} | \Psi_\mathrm{i} \rangle \,, \tag{S5}$$

where E_e is the photoelectron energy, $\vec{\mu} \cdot \vec{E}$ is the interaction term, $\Psi_{\vec{k}}$ is the wave function of photoelectron, and Ψ_i and Ψ_f represent the electronic wave functions of the initial and final states, respectively.

The full photoionization dipole matrix element $\mathcal{T}^{p_\mathrm{i}\mu_\mathrm{i},p_\mathrm{f}\mu_\mathrm{f}}_{\mu_0}(\theta_{\vec{k}}, \phi_{\vec{k}}, \theta_{\vec{e}_\mathrm{p}}, \phi_{\vec{e}_\mathrm{p}})$ is expanded in the radial part $\mathcal{I}^{p_\mathrm{i}\mu_\mathrm{i},p_\mathrm{f}\mu_\mathrm{f}}_{l,m,\mu}(E_\mathrm{e})$ and angular part (spherical harmonic functions and Wigner rotation matrix element),

$$\mathcal{T}^{p_\mathrm{i}\mu_\mathrm{i},p_\mathrm{f}\mu_\mathrm{f}}_{\mu_0}(\theta_{\vec{k}}, \phi_{\vec{k}}, \theta_{\vec{e}_\mathrm{p}}, \phi_{\vec{e}_\mathrm{p}}) = \mathcal{F}(E_\mathrm{e}) \sum_{l,m,\mu} \mathcal{I}^{p_\mathrm{i}\mu_\mathrm{i},p_\mathrm{f}\mu_\mathrm{f}}_{l,m,\mu}(E_\mathrm{e}) Y^*_{lm}(\theta_{\vec{k}}, \phi_{\vec{k}}) D^1_{\mu,\mu_0}(R_{\vec{e}_\mathrm{p}}) \,, \tag{S6}$$

where $\mathcal{F}(E_\mathrm{e})$ is the scale factor to square root of Mbarn, p_i and p_f are irreducible representation of the initial and final state, μ_i and μ_f are components of the initial and final state, μ is the projection of the lab frame (LF) polarization μ_0 into the molecule frame (MF), $Y^*_{lm}(\theta_{\vec{k}}, \phi_{\vec{k}})$ is the spherical harmonic function and $D^1_{\mu,\mu_0}(R_{\vec{e}_\mathrm{p}})$ is the Wigner rotation matrix element, which depends on the Euler angle that projects the polarization in LF to the MF (*61*). The radial part of the dipole matrix element $\mathcal{I}^{p_\mathrm{i}\mu_\mathrm{i},p_\mathrm{f}\mu_\mathrm{f}}_{l,m,\mu}(E_\mathrm{e})$ is

$$\mathcal{I}^{p_\mathrm{i}\mu_\mathrm{i},p_\mathrm{f}\mu_\mathrm{f}}_{l,m,\mu}(E_\mathrm{e}) = \langle \Psi^{p_\mathrm{i},\mu_\mathrm{i}}_\mathrm{i} | \vec{d}_\mu | \Psi^{p_\mathrm{f},\mu_\mathrm{f}}_\mathrm{f} \Psi_{\vec{k}} \rangle \,, \tag{S7}$$

where $\Psi_{\vec{k}}$ is the wave function of photoelectron and $\vec{d}_\mu$ is the dipole operator (*61*).

The full photoionization dipole matrix element $\mathcal{T}_{\mu_0}^{p_i\mu_i,p_f\mu_f}(\theta_{\vec{k}},\phi_{\vec{k}},\theta_{\vec{e}_p},\phi_{\vec{e}_p})$ is a complex number whose absolute value is the photoionization dipole amplitude and whose angle ϕ is the photoionization phase. The Wigner delay of photoionization τ_w is (*62, 63, 64*)

$$\tau_w = -\hbar\frac{d\phi}{dE_e}\,, \tag{S8}$$

where minus sign is because the full matrix element has the form $e^{-i\phi}$, and E_e is the photoelectron energy. Due to the short shape resonance interval in A', we use the finite difference (DF) method to calculate the ionization delay as follows

$$\tau_w^{(DF)} = -\hbar\frac{\Delta\phi}{\Delta E_e}\,. \tag{S9}$$

To compare with the experiment, the ionization delay of electrons emitted along the polarization axis is calculated.

Molecular frame photoelectron angular distributions (MFPADs) is obtained by the photoionization dipole amplitude (*64*),

$$\mathcal{I}_{\mu_0}(\theta_{\vec{k}},\phi_{\vec{k}},\theta_{\vec{e}_p},\phi_{\vec{e}_p}) = \sum_{\mu_i,\mu_f}|\mathcal{T}_{\mu_0}^{p_i\mu_i,p_f\mu_f}(\theta_{\vec{k}},\phi_{\vec{k}},\theta_{\vec{e}_p},\phi_{\vec{e}_p})|^2\,. \tag{S10}$$

In the partial wave calculation, the dipole matrix elements $\mathcal{T}_{\mu_0}^{p_i\mu_i,p_f\mu_f}(\theta_{\vec{k}},\phi_{\vec{k}},\theta_{\vec{e}_p},\phi_{\vec{e}_p})$ are expanded with spherical harmonic functions and the first 11 partial waves (l = 0-10) are calculated. The partial wave dipole matrix element labeled with angular quantum numbers l and m is

$$\mathcal{T}_{l,m,\mu_0}^{p_i\mu_i,p_f\mu_f}(\theta_{\vec{k}},\phi_{\vec{k}},\theta_{\vec{e}_p},\phi_{\vec{e}_p}) = \mathcal{F}(E_e)\sum_{\mu}\mathcal{I}_{l,m,\mu}^{p_i\mu_i,p_f\mu_f}(E_e)Y_{lm}^*(\theta_{\vec{k}},\phi_{\vec{k}})D_{\mu,\mu_0}^1(R_{\vec{e}_p})\,. \tag{S11}$$

Two-photon ionization delay of molecules with interchannel coupling

In the RABBIT measurement, the interaction of molecules with XUV and IR laser pulses undergoes the two-photon ionization process from the initial state (neutral ground state) to the final state (photoelectron continuum state and residual ion state). The two-photon ionization matrix element in the molecular frame with a fixed molecular orientation in space can be given by (*51*)

$$M(\vec{k};E_i+\Omega) = -i\int\frac{\langle\Psi_{f,\vec{k}}^{(-)}|\vec{r}\cdot\vec{E}_{IR}|\Psi_\nu\rangle\langle\Psi_\nu|\vec{r}\cdot\vec{E}_{XUV}|\Psi_i\rangle}{E_i+\Omega-E_\nu+i0}dE_\nu\,, \tag{S12}$$

where $\Psi^{(-)}_{f,\vec{k}}$ is the final state, Ψ_i is the initial state, Ψ_ν is the intermediate continuum state with energy E_ν, $\vec{k}$ represents the photoelectron momentum, E_i is the negative energy of initial state, Ω is the XUV photon energy, $\vec{E}_{\rm IR}$ denotes the electric field of the IR laser, and $\vec{E}_{\rm XUV}$ denotes the electric field of the XUV laser. After considering the partial-wave expansion and the asymptotic approximation, the above two-photon ionization matrix elements can be simplified to

$$M(\vec{k}; E_i + \Omega) = A_{\kappa k} \sum_{LM} b_{LM} Y_{LM}(\hat{k}) \,, \tag{S13}$$

where $A_{\kappa k}$ is the IR-induced two-photon amplitude, Y_{LM} denotes the spherical harmonic function, b_{LM} depends on orientation $\hat{R}_\gamma$ and its expression can be found in (*51*). And in our case, that is, in the case of linearly polarized light, $m_1 = 0$ and $m_2 = 0$. The single-photon ionization matrix element corresponding to the XUV-absorption is contained in b_{LM}. The residual ionic state after photoionization is not the single configuration after single-channel ionization, but rather the result of the coupling of multi-channel configurations. Therefore, we adopt the methods mentioned in (*46*) and (*23*) to handle the interchannel coupling. We use a close-coupling expansion to handle the scattering wave function, which contains the sum of the products of the residual ion state times the photoelectron wave function. We use the *Columbus* quantum chemistry program (*65, 66, 67*) to conduct multiconfiguration self-consistent field (MCSCF) calculations in the *aug-cc-pVTZ* basis set keeping the five orbitals with the lowest energy doubly occupied and obtained the ionic states.

In the finite-difference approximation, the two-photon ionization delay of a photoelectron sideband corresponding to energy $2q\omega$ is

$$\tau(2q, \hat{k}, \hat{R}_\gamma) = \frac{\arg(M^{(2q-1)*} M^{(2q+1)})}{2\omega} \,, \tag{S14}$$

which depends on the photoelectron emission angle and molecular orientation. The label $(2q \pm 1)$ indicate that the XUV laser energy used for ionizing the intermediate photoelectron state is $(2q \pm 1)\omega$. The delay can be split into the sum of two parts: photon energies-dependen continuum-continuum delay $\tau_{\rm cc}$ and molecular ionization delay $\tau_{\rm mol}$ as (*51*)

$$\tau_{\rm cc}(2q) = \frac{\arg(A^{(2q-1)*}_{\kappa_- k} A^{(2q+1)}_{\kappa_+ k})}{2\omega} \,, \tag{S15}$$

$$\tau_{\rm mol}(2q, \hat{k}, \hat{R}_\gamma) = \frac{\arg[\sum_{LM,L'M'} b^{(2q-1)*}_{L'M'} Y^*_{L'M'}(\hat{k}) b^{(2q+1)}_{LM} Y_{LM}(\hat{k})]}{2\omega} \,, \tag{S16}$$

Hamiltonian for the vibrational levels of CO_2^+ in the $A^2\Pi_u$ state with Renner-Teller effect

The CO_2 molecule, as a representative linear triatomic structure, exhibits three types of vibrational modes: symmetric stretching, antisymmetric stretching, and bending. The harmonic frequency for symmetric stretching is $\omega_g = 0.1675$ eV; for antisymmetric stretching $\omega_u = 0.2971$ eV; and for bending $\omega_\rho = 0.0833$ eV (*68*). In the $A^2\Pi_u$ state of CO_2^+, the primary contribution to the vibrational transition comes from the symmetric stretching mode and the bending mode (*45*), the antisymmetric stretching is left unexcited.

For the neutral CO_2 molecule, we treat it with a three-dimensional harmonic oscillator model containing three vibration modes: symmetric stretching, antisymmetric stretching and bending. For the $A^2\Pi_u$ state of CO_2^+, the Hamiltonian of the vibrational motion is

$$\mathcal{H} = (T + V_0)I + \Delta V \,, \tag{S17}$$

where T is the kinetic energy operator of the following form

$$T = -\frac{1}{2}\omega_g \frac{\partial^2}{\partial Q_g^2} - \frac{1}{2}\omega_u \frac{\partial^2}{\partial Q_u^2} - \frac{1}{2}\omega_\rho \frac{\partial^2}{\partial \rho^2} \,, \tag{S18}$$

V_0 is the harmonic oscillator potential energy operator,

$$V_0 = \frac{1}{2}\omega_g Q_g^2 + \frac{1}{2}\omega_u Q_u^2 + \frac{1}{2}\omega_\rho \rho^2 \,, \tag{S19}$$

ΔV is the modified potential energy matrix due to ionization and Renner-Teller effect,

$$\Delta V = \begin{pmatrix} E_{\pi_u} + \sqrt{2}\kappa Q_g + \Delta V' & \gamma\rho^2 \\ \gamma\rho^2 & E_{\pi_u} + \sqrt{2}\kappa Q_g + \Delta V' \end{pmatrix} , \tag{S20}$$

$$\Delta V' = 2\mu_g Q_g^2 + 2\mu_u Q_u^2 + 2\mu_\rho \rho^2 \,, \tag{S21}$$

and I is a 2×2 unit matrix. Q_g is the symmetric stretching coordinate,

$$Q_g = \frac{1}{\hbar}\sqrt{\frac{m_O \omega_g}{2}}(r_1 + r_2) \,, \tag{S22}$$

Q_u is the antisymmetric stretching coordinate,

$$Q_u = \frac{1}{\hbar}\sqrt{\frac{m_O m_C \omega_u}{2(2m_O + m_C)}}(r_1 - r_2) \,, \tag{S23}$$

and ρ is the bending coordinate,

$$\rho = \frac{a_e}{\hbar}\sqrt{\frac{\omega_\rho}{\frac{2}{m_O}(1+2\frac{m_O}{m_C})}}\Delta\theta\,, \tag{S24}$$

where m_O is the mass of oxygen atom, m_C is the mass of carbon atom, $\Delta\theta$ is the bending angle from linear configuration, a_e is C–O bond length in equilibrium configuration, r_1 and r_2 are the lengths of the two C-O bond, respectively. E_{π_u} is the ionization energy in equilibrium configuration, κ, μ_g, μ_u and μ_ρ are coupling constants in CO_2^+, γ is the Renner-Teller coupling constant (*68*).

We use the multi-configuration time-dependent Hartree (MCTDH) method (*52, 53, 54, 55*) to solve the time-dependent Schrödinger equations for the ground state of neutral CO_2 based on the three-dimensional harmonic oscillator Hamiltonian, and for the vibrational levels in $A^2\Pi_u$ based on the Hamiltonian as Eq. S17, and then obtain the vibrational wave functions.

Partial wave analysis of vibration states in the bent state and linear $A^2\Pi_u$ state

As a supplement to the origin of the shape resonance structure mentioned in the main text, we show the partial wave calculation results of the dipole matrix element of the state A' and A'' when the molecule is bent by 1° (see Fig. S6). The results show that the partial waves of the A'' state are obviously different from those of the A' state. In contrast, we calculate the ionization dipole matrix elements in the $A^2\Pi_u$ state of linear molecular configuration (see Fig. S7), and the results show that there is no shape resonance in the linear molecular configuration.

Wigner delay at different bending angles

As a supplement, we calculate the Wigner delays for CO_2^+ with several different bending angles. We take the bending angles as 0.5°, 1.5°, and 2° respectively (see Fig. S8). The results show that the resonance of the ionization delay becomes more pronounced with increasing bending angle.

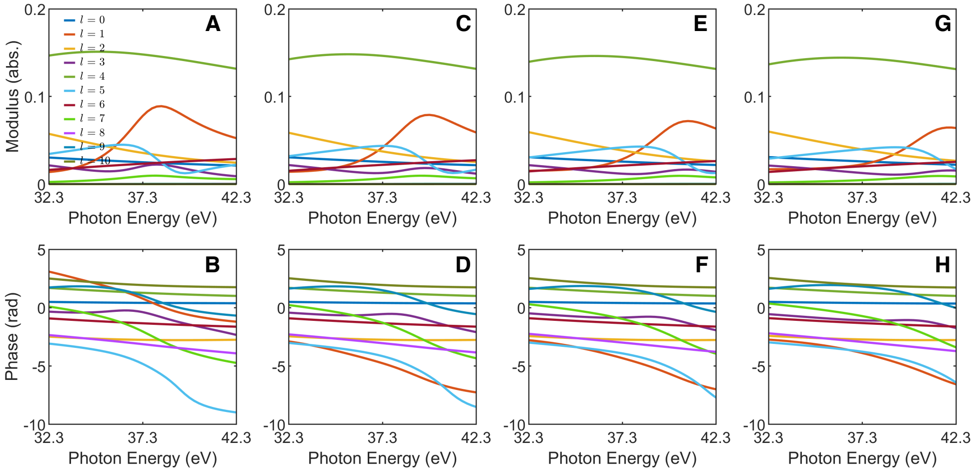

Figure S5: **| Partial wave analysis of vibration states of A' state.** The amplitude and phase of different partial waves (l = 0 to 10) for **(A-B)** R_{CO} = 1.158 Å of v = 0; **(C-D)** R_{CO} = 1.142 Å of v = 1; **(E-F)** R_{CO} = 1.131 Å of v = 2; **(G-H)** R_{CO} = 1.119 Å of v = 3, under the A' state with 1° bending angle.

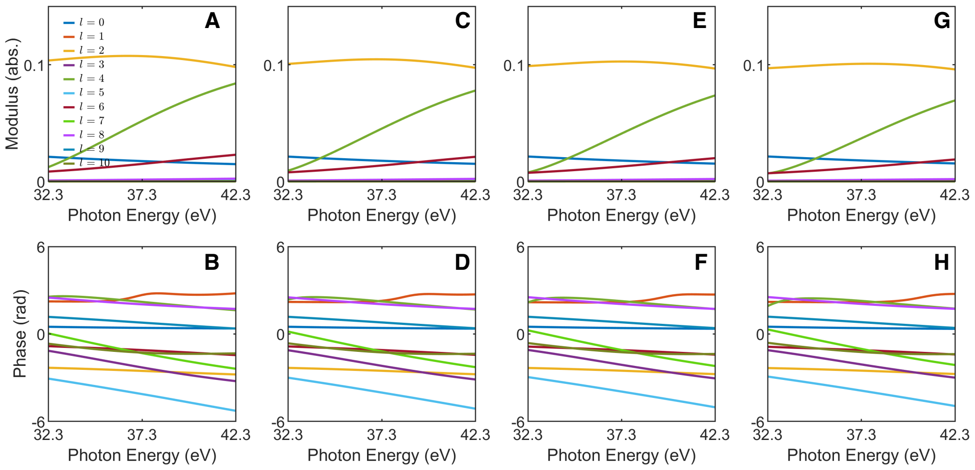

Figure S6: **Partial wave analysis of vibration states of A'' state.** The amplitude and phase of different partial waves (l = 0 to 10) for **(A-B)** R_{CO} = 1.158 Å of v = 0; **(C-D)** R_{CO} = 1.142 Å of v = 1; **(E-F)** R_{CO} = 1.131 Å of v = 2; **(G-H)** R_{CO} = 1.119 Å of v = 3, under the A'' state with 1° bending angle.

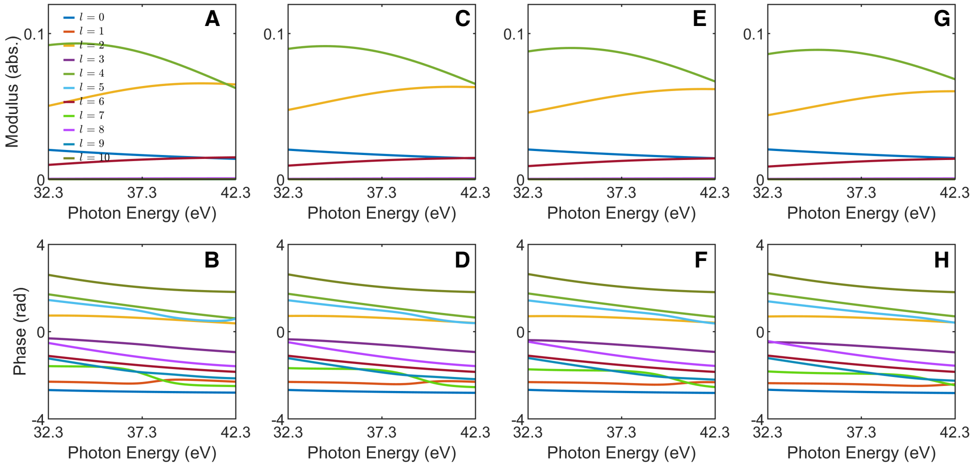

Figure S7: **Partial wave analysis of vibration states of $A^2\Pi_u$ state in linear molecular configuration.** The amplitude and phase of different partial waves (l = 0 to 10) for **(A-B)** R_{CO} = 1.158 Å of v = 0; **(C-D)** R_{CO} = 1.142 Å of v = 1; **(E-F)** R_{CO} = 1.131 Å of v = 2; **(G-H)** R_{CO} = 1.119 Å of v = 3, under the $A^2\Pi_u$ state in linear molecular configuration.

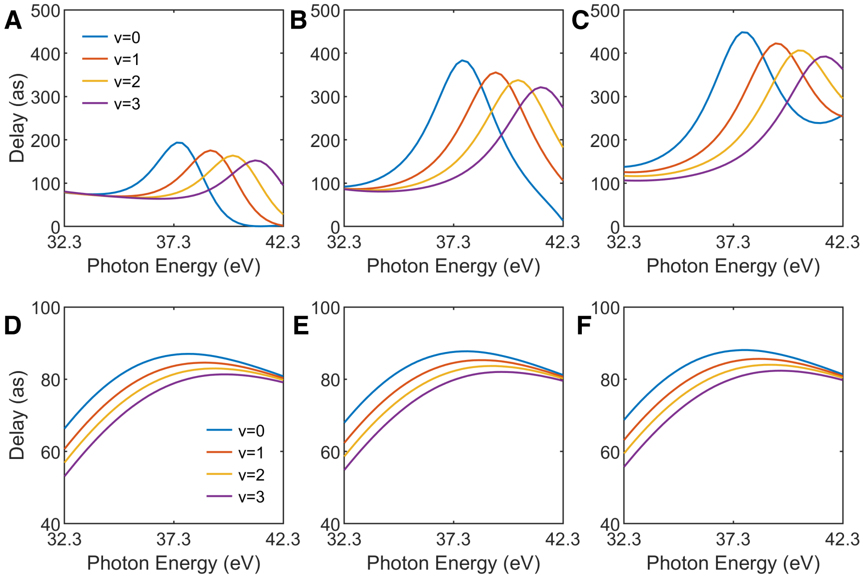

Figure S8: **Wigner time delay at different bending angles.** The vibration-dependent Wigner time delay of **(A-C)** A' state and **(D-F)** A'' state, with the bending angle from left to right are 0.5°, 1.5° and 2°, respectively. As the bending angle of the molecule increases, the Wigner delay caused by shape resonance in the A' state becomes larger, and the Wigner delays remain almost intact in the A'' state.

Influence of molecular orientation on Wigner delay

In the experiment, only photoelectrons emitted along the polarization axis of the light are collected by the spectrometer. Thus, ionization anisotropy and molecular alignment can influence the measured photoelectron spectrum. To account for orientation-dependent effects, the ionization probabilities as a function of molecular orientation can be computed using the method described in Ref. (*69*), thereby, obtaining the function of the ionization probability with relation to the orientation angle of the molecules. The relationship between the ionization probability and the orientation angle Θ can be simplified as

$$P(\Theta) \propto 1 + \beta P_2(\cos\Theta), \tag{S25}$$

where $\beta = 2$ corresponds to the parallel transition and $\beta = -1$ corresponds to the perpendicular transition. Experimental β parameters for photoelectrons associated with four final cationic states were reported in a previous study (*44*). For the $A^2\Pi_u$ state, β values are close to 1.5 in the photon energy range relevant to our experiment. Based on these values, we assumed that the molecular orientation is predominantly aligned along the laboratory-frame z-axis, corresponding to the polarization direction of the linearly polarized light. Our calculations also show that a deviation of the molecular axis from the z-axis by 5° and 10° does not substantially affect the results as shown in Fig. S9, and thus does not alter our main conclusions. Since β is photon-energy dependent, we did not include its variation explicitly in each Wigner delay calculation.

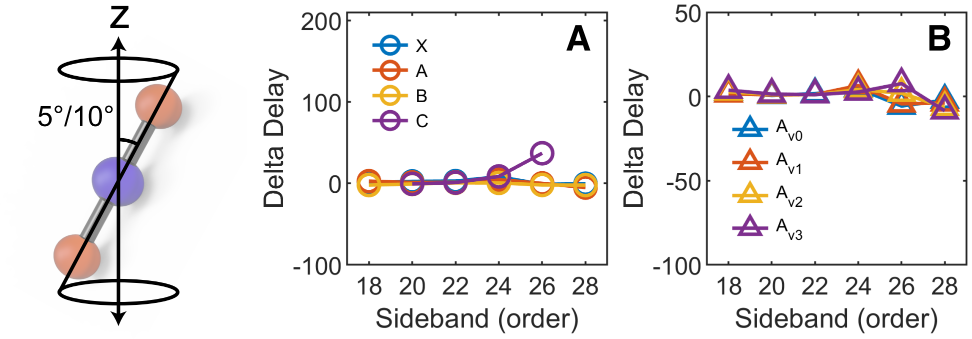

Figure S9: **Orientation dependent Wigner delay.** The difference of Wigner delay between orientation angles sets at 5° and 10° for (**A**) the electronic states and (**B**) the vibrational levels of $A^2\Pi_u$.

REFERENCES AND NOTES

1. P. W. Higgs, Broken symmetries and the masses of gauge bosons. *Phys. Rev. Lett.* **13**, 508–509 (1964).

2. R. Renner, Zur Theorie der Wechselwirkung zwischen Elektronen- und Kernbewegung bei dreiatomigen, stabförmigen Molekülen. *Z. Physik* **92**, 172–193 (1934).

3. H. A. Jahn, E. Teller, Stability of polyatomic molecules in degenerate electronic states - I - Orbital degeneracy. *Proc. R. Soc. Lond. A* **161**, 220–235 (1937).

4. H. J. Wörner, J. B. Bertrand, B. Fabre, J. Higuet, H. Ruf, A. Dubrouil, S. Patchkovskii, M. Spanner, Y. Mairesse, V. Blanchet, E. Mével, E. Constant, P. B. Corkum, D. M. Villeneuve, Conical intersection dynamics in NO_2 probed by homodyne high-harmonic spectroscopy. *Science* **334**, 208–212 (2011).

5. K. S. Zinchenko, F. Ardana-Lamas, I. Seidu, S. P. Neville, J. van der Veen, V. U. Lanfaloni, M. S. Schuurman, H. J. Wörner, Sub-7-femtosecond conical-intersection dynamics probed at the carbon K-edge. *Science* **371**, 489–494 (2021).

6. T. Schultz, E. Samoylova, W. Radloff, I. V. Hertel, A. L. Sobolewski, W. Domcke, Efficient deactivation of a model base pair via excited-state hydrogen transfer. *Science* **306**, 1765–1768 (2004).

7. A. L. Sobolewski, W. Domcke, C. Hättig, Tautomeric selectivity of the excited-state lifetime of guanine/cytosine base pairs: The role of electron-driven proton-transfer processes. *Proc. Natl. Acad. Sci. U.S.A.* **102**, 17903–17906 (2005).

8. K. Amini, M. Sclafani, T. Steinle, A.-T. Le, A. Sanchez, C. Müller, J. Steinmetzer, L. Yue, J. R. M. Saavedra, M. Hemmer, M. Lewenstein, R. Moshammer, T. Pfeifer, M. G. Pullen, J. Ullrich, B. Wolter, R. Moszynski, F. J. G. de Abajo, C. D. Lin, S. Gräfe, J. Biegert, Imaging the Renner–Teller effect using laser-induced electron diffraction. *Proc. Natl. Acad. Sci. U.S.A.* **116**, 8173–8177 (2019).

9. M. Li, M. Zhang, O. Vendrell, Z. Guo, Q. Zhu, X. Gao, L. Cao, K. Guo, Q.-Q. Su, W. Cao, S. Luo, J. Yan, Y. Zhou, Y. Liu, Z. Li, P. Lu, Ultrafast imaging of spontaneous symmetry breaking in a photoionized molecular system. *Nat. Commun.* **12**, 4233 (2021).

10. E. Ridente, D. Hait, E. A. Haugen, A. D. Ross, D. M. Neumark, M. Head-Gordon, S. R. Leone, Femtosecond symmetry breaking and coherent relaxation of methane cations via x-ray spectroscopy. *Science* **380**, 713–717 (2023).

11. K. S. Zinchenko, F. Ardana-Lamas, V. U. Lanfaloni, N. Monahan, I. Seidu, M. S. Schuurman, S. P. Neville, H. J. Wörner, Few-femtosecond electronic and structural rearrangements of CH_4^+ driven by the Jahn–Teller effect. *Struct. Dyn.* **10**, 064303 (2023).

12. M. Ferray, A. L'Huillier, X. F. Li, L. A. Lompre, G. Mainfray, C. Manus, Multiple-harmonic conversion of 1064 nm radiation in rare gases. *J. Phys. B At. Mol. Opt. Phys.* **21**, L31 (1987).

13. P. M. Paul, E. S. Toma, P. Breger, G. Mullot, F. Augé, P. Balcou, H. G. Muller, P. Agostini, Observation of a train of attosecond pulses from high harmonic generation. *Science* **292**, 1689–1692 (2001).

14. M. Hentschel, R. Kienberger, C. Spielmann, G. A. Reider, N. Milosevic, T. Brabec, P. Corkum, U. Heinzmann, M. Drescher, F. Krausz, Attosecond metrology. *Nature* **414**, 509–513 (2001).

15. E. Goulielmakis, Z.-H. Loh, A. Wirth, R. Santra, N. Rohringer, V. S. Yakovlev, S. Zherebtsov, T. Pfeifer, A. M. Azzeer, M. F. Kling, S. R. Leone, F. Krausz, Real-time observation of valence electron motion. *Nature* **466**, 739–743 (2010).

16. V. Gruson, L. Barreau, Á. Jiménez-Galan, F. Risoud, J. Caillat, A. Maquet, B. Carré, F. Lepetit, J.-F. Hergott, T. Ruchon, L. Argenti, R. Taïeb, F. Martín, P. Salières, Attosecond dynamics through a Fano resonance: Monitoring the birth of a photoelectron. *Science* **354**, 734–738 (2016).

17. M. Isinger, R. J. Squibb, D. Busto, S. Zhong, A. Harth, D. Kroon, S. Nandi, C. L. Arnold, M. Miranda, J. M. Dahlström, E. Lindroth, R. Feifel, M. Gisselbrecht, A. L'Huillier, Photoionization in the time and frequency domain. *Science* **358**, 893–896 (2017).

18. M. Li, M.-F. Xie, H. Wang, L. Jia, J. Li, W. Wang, J. Cai, X. Hong, X. Shi, Y. Lv, X. Zhao, S. Luo, W.-C. Jiang, L.-Y. Peng, D. Ding, Observation of laser-assisted dynamic interference by attosecond controlled photoelectron spectroscopy. *Phys. Rev. Lett.* **133**, 253201 (2024).

19. M. Huppert, I. Jordan, D. Baykusheva, A. von Conta, H. J. Wörner, Attosecond delays in molecular photoionization. *Phys. Rev. Lett.* **117**, 093001 (2016).

20. S. Nandi, E. Plésiat, S. Zhong, A. Palacios, D. Busto, M. Isinger, L. Neoričić, C. L. Arnold, R. J. Squibb, R. Feifel, P. Decleva, A. L'Huillier, F. Martín, M. Gisselbrecht, Attosecond timing of electron emission from a molecular shape resonance. *Sci. Adv.* **6**, eaba7762 (2020).

21. S. Heck, D. Baykusheva, M. Han, J.-B. Ji, C. Perry, X. Gong, H. J. Wörner, Attosecond interferometry of shape resonances in the recoil frame of CF_4. *Sci. Adv.* **7**, eabj8121 (2021).

22. V. J. Borràs, J. González-Vázquez, L. Argenti, F. Martín, Attosecond photoionization delays in the vicinity of molecular Feshbach resonances. *Sci. Adv.* **9**, eade3855 (2023).

23. D. Hammerland, T. Berglitsch, P. Zhang, T. T. Luu, K. Ueda, R. R. Lucchese, H. J. Wörner, Bond-length dependence of attosecond ionization delays in O_2 arising from electron correlation to a shape resonance. *Sci. Adv.* **10**, eadl3810 (2024).

24. S. Li, T. Driver, P. Rosenberger, E. G. Champenois, J. Duris, A. Al-Haddad, V. Averbukh, J. C. T. Barnard, N. Berrah, C. Bostedt, P. H. Bucksbaum, R. N. Coffee, L. F. DiMauro, L. Fang, D. Garratt, A. Gatton, Z. Guo, G. Hartmann, D. Haxton, W. Helml, Z. Huang, A. C. LaForge, A. Kamalov, J. Knurr, M.-F. Lin, A. A. Lutman, J. P. MacArthur, J. P. Marangos, M. Nantel, A. Natan, R. Obaid, J. T. O'Neal, N. H. Shivaram, A. Schori, P. Walter, A. L. Wang, T. J. A. Wolf, Z. Zhang, M. F. Kling, A. Marinelli, J. P. Cryan, Attosecond coherent electron motion in Auger-Meitner decay. *Science* **375**, 285–290 (2022).

25. J. Vos, L. Cattaneo, S. Patchkovskii, T. Zimmermann, C. Cirelli, M. Lucchini, A. Kheifets, A. S. Landsman, U. Keller, Orientation-dependent stereo Wigner time delay and electron localization in a small molecule. *Science* **360**, 1326–1330 (2018).

26. H. Ahmadi, E. Plésiat, M. Moioli, F. Frassetto, L. Poletto, P. Decleva, C. Schröter, T. Pfeifer, R. Moshammer, A. Palacios, F. Martin, G. Sansone, Attosecond photoionisation time delays reveal the anisotropy of the molecular potential in the recoil frame. *Nat. Commun.* **13**, 1242 (2022).

27. T. Driver, M. Mountney, J. Wang, L. Ortmann, A. Al-Haddad, N. Berrah, C. Bostedt, E. G. Champenois, L. F. DiMauro, J. Duris, D. Garratt, J. M. Glownia, Z. Guo, D. Haxton, E. Isele, I. Ivanov, J. Ji, A. Kamalov, S. Li, M.-F. Lin, J. P. Marangos, R. Obaid, J. T. O'Neal, P. Rosenberger, N. H. Shivaram, A. L. Wang, P. Walter, T. J. A. Wolf, H. J. Wörner, Z. Zhang, P. H. Bucksbaum, M. F. Kling, A. S. Landsman, R. R. Lucchese, A. Emmanouilidou, A. Marinelli, J. P. Cryan, Attosecond delays in x-ray molecular ionization. *Nature* **632**, 762–767 (2024).

28. I. Jordan, M. Huppert, D. Rattenbacher, M. Peper, D. Jelovina, C. Perry, A. von Conta, A. Schild, H. J. Wörner, Attosecond spectroscopy of liquid water. *Science* **369**, 974–979 (2020).

29. S. Li, L. Lu, S. Bhattacharyya, C. Pearce, K. Li, E. T. Nienhuis, G. Doumy, R. D. Schaller, S. Moeller, M.-F. Lin, G. Dakovski, D. J. Hoffman, D. Garratt, K. A. Larsen, J. D. Koralek, C. Y. Hampton, D. Cesar, J. Duris, Z. Zhang, N. Sudar, J. P. Cryan, A. Marinelli, X. Li, L. Inhester, R. Santra, L. Young, Attosecond-pump attosecond-probe x-ray spectroscopy of liquid water. *Science* **383**, 1118–1122 (2024).

30. A. Sommer, E. M. Bothschafter, S. A. Sato, C. Jakubeit, T. Latka, O. Razskazovskaya, H. Fattahi, M. Jobst, W. Schweinberger, V. Shirvanyan, V. S. Yakovlev, R. Kienberger, K. Yabana, N. Karpowicz, M. Schultze, F. Krausz, Attosecond nonlinear polarization and light–matter energy transfer in solids. *Nature* **534**, 86–90 (2016).

31. H. Y. Kim, M. Garg, S. Mandal, L. Seiffert, T. Fennel, E. Goulielmakis, Attosecond field emission. *Nature* **613**, 662–666 (2023).

32. P. Dienstbier, L. Seiffert, T. Paschen, A. Liehl, A. Leitenstorfer, T. Fennel, P. Hommelhoff, Tracing attosecond electron emission from a nanometric metal tip. *Nature* **616**, 702–706 (2023).

33. M. Ossiander, F. Siegrist, V. Shirvanyan, R. Pazourek, A. Sommer, T. Latka, A. Guggenmos, S. Nagele, J. Feist, J. Burgdörfer, R. Kienberger, M. Schultze, Attosecond correlation dynamics. *Nat. Phys.* **13**, 280–285 (2017).

34. S. Biswas, B. Förg, L. Ortmann, J. Schötz, W. Schweinberger, T. Zimmermann, L. Pi, D. Baykusheva, H. A. Masood, I. Liontos, A. M. Kamal, N. G. Kling, A. F. Alharbi, M. Alharbi, A. M. Azzeer, G. Hartmann, H. J. Wörner, A. S. Landsman, M. F. Kling, Probing molecular environment through photoemission delays. *Nat. Phys.* **16**, 778–783 (2020).

35. V. Loriot, A. Boyer, S. Nandi, C. M. González-Collado, E. Plésiat, A. Marciniak, C. L. Garcia, Y. Hu, M. Lara-Astiaso, A. Palacios, P. Decleva, F. Martín, F. Lépine, Attosecond metrology of the two-dimensional charge distribution in molecules. *Nat. Phys.* **20**, 765–769 (2024).

36. M. Nisoli, P. Decleva, F. Calegari, A. Palacios, F. Martín, Attosecond electron dynamics in molecules. *Chem. Rev.* **117**, 10760–10825 (2017).

37. F. Calegari, F. Martin, Open questions in attochemistry. *Commun. Chem.* **6**, 184 (2023).

38. H. Yoshida, K. Nobusada, K. Okada, S. Tanimoto, N. Saito, A. De Fanis, K. Ueda, Symmetry-resolved vibrational spectroscopy for the C $1s^{-1}2\pi_u$ Renner-Teller pair states in CO_2. *Phys. Rev. Lett.* **88**, 083001 (2002).

39. Y. Muramatsu, K. Ueda, N. Saito, H. Chiba, M. Lavollée, A. Czasch, T. Weber, O. Jagutzki, H. Schmidt-Böcking, R. Moshammer, U. Becker, K. Kubozuka, I. Koyano, Direct probe of the bent and linear geometries of the core-excited Renner-Teller pair states by means of the triple-ion-coincidence momentum imaging technique. *Phys. Rev. Lett.* **88**, 133002 (2002).

40. T. Tanaka, C. Makochekanwa, H. Tanaka, M. Kitajima, M. Hoshino, Y. Tamenori, E. Kukk, X. J. Liu, G. Prümper, K. Ueda, Symmetry-resolved absorption spectra of vibrationally excited CO_2 molecules. *Phys. Rev. Lett.* **95**, 203002 (2005).

41. A. Potts, G. Fattahallah, High-resolution ultraviolet photoelectron spectroscopy of CO_2, COS and CS_2. *J. Phys. B At. Mol. Opt. Phys.* **13**, 2545–2556 (1980).

42. P. Roy, I. Nenner, P. Millie, P. Morin, D. Roy, Experimental and theoretical study of configuration interaction states of CO_2^+. *J. Chem. Phys.* **84**, 2050–2061 (1986).

43. R. R. Lucchese, Effects of interchannel coupling on the photoionization cross sections of carbon dioxide. *J. Chem. Phys.* **92**, 4203–4211 (1990).

44. M. R. F. Siggel, J. B. West, M. A. Hayes, A. C. Parr, J. L. Dehmer, I. Iga, Shape-resonance-enhanced continuum–continuum coupling in photoionization of CO_2. *J. Chem. Phys.* **99**, 1556–1563 (1993).

45. P. Baltzer, F. T. Chau, J. H. D. Eland, L. Karlsson, M. Lundqvist, J. Rostas, K. Y. Tam, H. Veenhuizen, B. Wannberg, A study of the vibronic structure in the HeI excited photoelectron spectrum of CO_2 involving the $X^2\Pi_g$ and $A^2\Pi_u$ ionic states. *J. Chem. Phys.* **104**, 8922–8931 (1996).

46. A. Kamalov, A. L. Wang, P. H. Bucksbaum, D. J. Haxton, J. P. Cryan, Electron correlation effects in attosecond photoionization of CO_2. *Phys. Rev. A* **102**, 023118 (2020).

47. J. Benda, Z. Mašín, J. D. Gorfinkiel, Analysis of RABITT time delays using the stationary multiphoton molecular *R*-matrix approach. *Phys. Rev. A* **105**, 053101 (2022).

48. V. Véniard, R. Taïeb, A. Maquet, Phase dependence of $(N + 1)$-color $(N > 1)$ IR-UV photoionization of atoms with higher harmonics. *Phys. Rev. A* **54**, 721–728 (1996).

49. F. A. Gianturco, R. R. Lucchese, N. Sanna, Calculation of low-energy elastic cross sections for electron-CF_4 scattering. *J. Chem. Phys.* **100**, 6464–6471 (1994).

50. A. P. P. Natalense, R. R. Lucchese, Cross section and asymmetry parameter calculation for sulfur 1s photoionization of SF_6. *J. Chem. Phys.* **111**, 5344–5348 (1999).

51. D. Baykusheva, H. J. Wörner, Theory of attosecond delays in molecular photoionization. *J. Chem. Phys.* **146**, 124306 (2017).

52. G. A. Worth, M. H. Beck, A. Jäckle, H.-D. Meyer, The MCTDH Package, Version 8.5, (2021), http://mctdh.uni-hd.de.

53. M. H. Beck, A. Jäckle, G. A. Worth, H.-D. Meyer, The multiconfiguration time-dependent Hartree (MCTDH) method: A highly efficient algorithm for propagating wavepackets. *Phys. Rep.* **324**, 1–105 (2000).

54. H.-D. Meyer, U. Manthe, L. S. Cederbaum, The multi-configurational time-dependent Hartree approach. *Chem. Phys. Lett.* **165**, 73–78 (1990).

55. U. Manthe, H.-D. Meyer, L. S. Cederbaum, Wave-packet dynamics within the multiconfiguration Hartree framework: General aspects and application to NOCl. *J. Chem. Phys.* **97**, 3199–3213 (1992).

56. M. Li, H. Wang, X. Li, J. Wang, J. Zhang, X. San, P. Ma, Y. Lu, Z. Liu, C. Wang, Y. Yang, S. Luo, D. Ding, Stable attosecond beamline equipped with high resolution electron and XUV spectrometer based on high-harmonics generation. *J. Electron. Spectrosc. Relat. Phenom.* **263**, 147287 (2023).

57. I. Jordan, H. J. Wörner, Extracting attosecond delays from spectrally overlapping interferograms. *J. Opt.* **20**, 024013 (2018).

58. H.-J. Werner, P. J. Knowles, G. Knizia, F. R. Manby, M. Schütz, Molpro: A general-purpose quantum chemistry program package. *WIREs Comput. Mol. Sci.* **2**, 242–253 (2012).

59. R. Polák, M. Hochlaf, M. Levinas, G. Chambaud, P. Rosmus, On the potential energy functions of the electronic states of CO_2^+. *Spectrochim Acta A Mol Biomol Spectrosc.* **55**, 447–456 (1999).

60. S. Larimian, S. Erattupuzha, S. Mai, P. Marquetand, L. González, A. Baltuška, M. Kitzler, X. Xie, Molecular oxygen observed by direct photoproduction from carbon dioxide. *Phys. Rev. A* **95**, 011404 (2017).

61. D. Toffoli, R. R. Lucchese, M. Lebech, J. C. Houver, D. Dowek, Molecular frame and recoil frame photoelectron angular distributions from dissociative photoionization of NO_2. *J. Chem. Phys.* **126**, 054307 (2007).

62. E. P. Wigner, Lower limit for the energy derivative of the scattering phase shift. *Phys. Rev.* **98**, 145–147 (1955).

63. F. T. Smith, Lifetime matrix in collision theory. *Phys. Rev.* **118**, 349–356 (1960).

64. J. M. Dahlström, A. L'Huillier, A. Maquet, Introduction to attosecond delays in photoionization. *J. Phys. B At. Mol. Opt. Phys.* **45**, 183001 (2012).

65. H. Lischka, T. Müller, P. G. Szalay, I. Shavitt, R. M. Pitzer, R. Shepard, Columbus–A program system for advanced multireference theory calculations. *WIREs Comput. Mol. Sci.* **1**, 191–199 (2011).

66. H. Lischka, R. Shepard, R. Pitzer, I. Shavitt, M. Dallos, T. Müller, P. Szalay, M. Seth, G. Kedziora, S. Yabushita, Z. Zhang, High-level multireference methods in the quantum-chemistry program system COLUMBUS: Analytic MR-CISD and MR-AQCC gradients and MR-AQCC-LRT for excited states, GUGA spin-orbit CI and parallel CI density. *Phys. Chem. Chem. Phys.* **3**, 664–673 (2001).

67. H. Lischka, R. Shepard, I. Shavitt, R. M. Pitzer, T. Müller, P. G. Szalay, S. R. Brozell, G. Kedziora, E. A. Stahlberg, R. J. Harrison, J. Nieplocha, M. Minkoff, M. Barbatti, M. Schuurmann, Y. Guan, D. R. Yarkony, S. Matsika, F. Plasser, E. V. Beck, J.-P. Blaudeau, M. Ruckenbauer, B. Sellner, J. Szymczak, R. F. K. Spada, A. Das, L. T. Belcher, R. Nieman, COLUMBUS, an ab initio electronic structure program, release 7.2 (2022); https://columbus-program-system.gitlab.io/columbus/.

68. T. Zimmermann, H. Köppel, L. S. Cederbaum, On the bilinear vibronic coupling mechanism. *J. Chem. Phys.* **83**, 4697–4709 (1985).

69. H. Timmers, Z. Li, N. Shivaram, R. Santra, O. Vendrell, A. Sandhu, Coherent electron hole dynamics near a conical intersection. *Phys. Rev. Lett.* **113**, 113003 (2014).
